# Exploring the Role of Polyunsaturated Fatty Acids in Children’s Sleep

**DOI:** 10.3390/biomedicines13092045

**Published:** 2025-08-22

**Authors:** Liuyan Zhu, Bingquan Zhu, Dan Yao

**Affiliations:** 1 Department of Pediatric Health Care, Children’s Hospital, Zhejiang University School of Medicine, National Clinical Research Center for Child Health, Hangzhou 310052, China; 21418239@zju.edu.cn (L.Z.); zhubingquan@zju.edu.cn (B.Z.); 2 Zhejiang Key Laboratory of Neonatal Diseases, Hangzhou 310052, China

**Keywords:** polyunsaturated fatty acids, docosahexaenoic acid, eicosapentaenoic acid, children, sleep

## Abstract

Research on the effects of polyunsaturated fatty acids on children’s sleep has made significant advancements. This study explores the unique pathways through which polyunsaturated fatty acids, particularly docosahexaenoic acid and eicosapentaenoic acid from the n-3 series, influence sleep regulation in children. Neurobiologically, docosahexaenoic acid and eicosapentaenoic acid have been shown to bi-directionally modulate neurotransmitters and circadian rhythms via the gut–brain axis, reshaping gut microbiota and affecting brain signaling. In terms of inflammation and immune regulation, this study is the first to confirm that Maresin1, produced from n-3 fatty acids, can inhibit the activation of specific inflammasomes, thereby mitigating the disruptive effects of pro-inflammatory cytokines on sleep. The analysis of clinical applications indicates that newly developed medium- and long-chain triglyceride formulations rich in docosahexaenoic acid exhibit excellent digestive absorption in infants’ gastrointestinal systems, paving the way for new products designed to enhance infant sleep. However, current research has limitations concerning the precise dosing of docosahexaenoic acid, the representativeness of samples, and the overall rigor of study designs. Mechanistically, polyunsaturated fatty acids may exert their effects through various pathways, including neurobiology, inflammation, immune regulation, and endocrine modulation. In clinical studies, different formulations of fish oil show varying safety profiles and bioavailability. Future research should prioritize high-quality studies to clarify how different doses of polyunsaturated fatty acids affect children’s sleep, assess long-term safety, and investigate interactions with other factors, ultimately providing solid theoretical and practical guidance for improving children’s sleep.

## 1. Introduction

Sleep is essential for physical and mental health, as well as the growth and development of children. Nonetheless, sleep disturbances are quite common in this population, affecting approximately 20% to 30% of children. Moreover, between 10% and 20% of these children suffer from sleep disorders that require medical attention [1,2]. Insufficient sleep impairs children’s cognitive functions, such as attention, memory, and learning, while also negatively impacting emotional regulation and immune function. Quality sleep is vital for normal brain development, as it supports neuronal growth and connectivity, creating a solid foundation for future educational and life prospects. For example, children who fail to obtain adequate sleep frequently find it challenging to concentrate in school, resulting in a diminished academic performance [3]. Extended sleep disturbances are associated with an increased risk of anxiety and depression [4]. A four-year longitudinal study indicated that preschoolers experiencing persistent sleep issues face a greater likelihood of anxiety during their school years [5]. Additionally, a comprehensive follow-up analysis of 2852 children revealed a notable link between sleep difficulties at age two and subsequent anxiety at age eight [6].

Among the various factors affecting children’s sleep quality, polyunsaturated fatty acids (PUFAs), especially docosahexaenoic acid (DHA, C22:6, n-3), have attracted increasing research attention. DHA is a key component of the omega-3 series of PUFAs, predominantly found in marine fish, and serves as an important structural element in both the brain and retina. Research shows that DHA plays a crucial role in regulating melatonin and serotonin production, which are vital for maintaining normal cellular structure in the brain and are closely associated with the sleep–wake cycle [7,8]. In a study of 405 Mexican adolescents, researchers found that higher plasma levels of DHA were associated with longer sleep durations [9]. Elevated concentrations of DHA correlated with an improved sleep quality and a lower risk of obstructive sleep apnea hypopnea syndrome (OSAHS) [10]. In another investigation conducted in the UK, researchers identified a positive correlation between plasma DHA levels and sleep quality, demonstrating that DHA supplementation significantly increased sleep duration and reduced the number of awakenings during the night [11]. Moreover, findings from the National Health and Nutrition Examination Survey (2011–2012; *N* = 1314) pointed to an association between omega-3 fatty acid levels and an adequate sleep duration, indicating that individuals averaging less than five hours of sleep per night consistently displayed lower levels of eicosapentaenoic acid (EPA, C20:5, and n-3), DHA, and total omega-3 fatty acids [12]. Conversely, a study by Boone KM et al. involving high-risk premature infants exhibiting autism spectrum disorder-related symptoms indicated that, while a 90-day regimen of omega 3-6-9 fatty acid supplements effectively reduced anxiety, depression, and internalizing behaviors—and improved social skills—no significant changes were noted in other behavioral or sleep outcomes [13]. Numerous studies have established a strong association between the consumption of fish and shellfish and sleep health [14,15]. Research by Patan et al. revealed that foods high in DHA and EPA significantly enhance sleep efficiency, reduce sleep onset latency, and improve overall sleep quality, potentially decreasing the risk of sleep disorders and insomnia [16]. Nevertheless, results across various studies can be inconsistent. For instance, Hysing M et al. reported no significant differences in sleep onset latency, waking times, or sleep duration between those consuming fish and those in a meat control group [17]. Additionally, a systematic review and meta-analysis revealed that omega-3 long-chain PUFAs might enhance sleep structure in infants and lower sleep disorder scores in children with clinical sleep problems, but they seem to have little effect on the sleep patterns of healthy children and adults [18].

**Figure 1:**
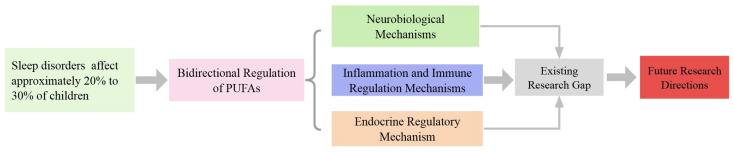
Sleep problems are common among children and can have detrimental effects on their learning, cognition, and memory. Research suggests that PUFAs may contribute to sleep improvement. However, their specific dual regulatory functions and underlying mechanisms primarily encompass three areas: neurobiological mechanisms, inflammation and immune regulation, and endocrine regulation. Despite these findings, several gaps remain in the current literature that warrant further exploration, particularly regarding the dosage, duration, and safety of PUFAs when utilized for enhancing sleep in children.

In summary, considerable uncertainty remains regarding the specific impacts of different doses of DHA on children’s sleep, as well as its interactions with other nutritional elements and environmental factors Additionally, concerns about the long-term safety of DHA supplementation continue to be a topic of discussion. This highlights a vital area for future research aimed at clarifying the intricate relationship between PUFAs and children’s sleep patterns. The ultimate aim is to fill existing research gaps and develop a solid theoretical basis for enhancing sleep quality in children. To facilitate the comprehension of the article’s overall framework, we have included a summary in Figure 1.

## 2. Concise Overview of Polyunsaturated Fatty Acids

PUFAs are a class of straight-chain fatty acids characterized by the presence of two or more unsaturated bonds, typically comprising carbon chains ranging from 18 to 22 carbons in length. PUFAs are classified into two primary families based on the position of the first unsaturated bond: n-3 and n-6 fatty acids. The n-3 family includes alpha-linolenic acid (α-LA, C18:3, and n-3), EPA, and DHA [19], which are deemed essential nutrients since the human body cannot synthesize them [20]. The n-6 family primarily consists of linoleic acid (LA, C18:2, and n-6), which serves as a precursor for the synthesis of gamma-linolenic acid and arachidonic acid (AA, C20:4). Notably, α-LA functions as a precursor for the n-3 series, undergoing enzymatic processes to form EPA and DHA. Significant sources of n-3 fatty acids include marine fish such as mackerel, salmon, herring, and sardines. The EPA and DHA derived from these fish are vital for various aspects of neurological health, encompassing synaptic formation, gene regulation, neural adaptability, membrane fluidity, neurotransmitter release, and myelination [21]. In contrast, LA is commonly found in Western diets, predominantly derived from oils like soybean, corn, and sunflower. AA, mainly sourced from peanut oil, animal products, eggs, and dairy, is essential for the structural integrity of cell membranes. The metabolites of AA are essential for maintaining membrane fluidity, enabling cellular communication, and supporting immune responses [22]. However, increased levels of AA, especially during oxidative stress, may contribute to negative outcomes such as insulin resistance and apoptosis, linking them to cardiovascular and metabolic diseases.

PUFAs account for 35% and 60% of the lipids in the adult brain, with EPA and DHA being essential for both structural and functional brain development [23]. DHA is the predominant structural fatty acid during brain maturation, notably accumulating in the later stages of pregnancy [24]. Although the brain can synthesize some unsaturated fatty acids, it does so at a rate that is inadequate to fulfill its requirements, leading to a reliance on PUFAs present in the bloodstream. The benefits of n-3 fatty acids include supporting brain maturation, enhancing cardiovascular health, improving mental wellness, and increasing cognitive performance [23,25]. DHA is particularly important for neuronal growth, synaptic flexibility, and the construction of neural membranes. In contrast, n-6 fatty acids are significant in regulating inflammatory responses and can induce dynamic modifications in sleep-related compounds, including endocannabinoids, lipid A, and prostaglandins [26]. Animal studies indicate that prostaglandins D2 and E2, derived from arachidonic acid, are crucial regulators of sleep [27].

Research suggests a correlation between the plasma levels of DHA and sleep duration. PUFAs support neuronal growth and repair by enhancing the properties of neuronal cell membranes, which in turn improves cognitive function. Additionally, n-3 fatty acids possess anti-inflammatory properties that reduce pro-inflammatory factor expression and enhance inflammatory responses within the brain [28,29]. In contrast, n-6 fatty acids are associated with pro-inflammatory effects, highlighting the importance of maintaining a balance between these two fatty acid types for optimal brain health. Dietary intake of PUFAs has been linked to various mental health issues, including attention deficit hyperactivity disorder, depression, and neurodegenerative diseases. Adequate supplementation of n-3 fatty acids may lower the risk of chronic inflammation-related diseases in the brain and enhance cognitive function. Animal studies have shown that DHA and EPA significantly improve glucose uptake efficiency and mitigate insulin resistance [30]. The underlying mechanisms may involve an increased activity of GPR40 and GPR120, promoting glucagon-like peptide release and enhancing insulin secretion while inhibiting inflammatory factors such as IL-6 and TNF-α. This regulation of hepatic lipid metabolism helps to reduce lipotoxicity. Human studies indicate that fish oil can modulate lipid metabolism, decrease oxidative stress, suppress inflammation, and enhance processes such as lipolysis and autophagy, all while improving antioxidant capacity and gut permeability [31]. These findings open avenues for further research into the relationship between PUFAs and sleep, advancing our understanding of their biological roles.

It is important to note that PUFAs play a significant role in maternal–fetal programming [32]. Maternal intake of n-6 fatty acids exhibits an inverted U-shaped relationship with offspring birth weight, where both excessively high and low intakes can lead to a low birth weight and hinder postnatal growth [33]. Animal studies have demonstrated that n-3 fatty acid supplementation shifts the sex ratio of offspring toward males, while diets rich in n-6 fatty acids tend to skew toward female offspring [34]. This phenomenon may be related to inflammation induced by prostaglandins; specifically, elevated prostaglandin E or low leptin levels during early pregnancy may contribute to male embryo loss [35]. Recent research has shown that a combination of n-3 fatty acids, gamma-linolenic acid, and vitamin D effectively alleviates symptoms of childhood atopic dermatitis [36], indicating that this combination is a safe and effective intervention for reducing dermatitis severity.

In conclusion, PUFAs significantly influence the brain, impacting nervous system development, functional maintenance, inflammation regulation, and cardiovascular health. Proper PUFAs intake is essential not only for brain health but also for improving sleep quality, regulating emotions, and enhancing cognitive functions. We have summarized the main classifications, sources, functions, and health associations of PUFAs in Table 1.

## 3. Current Research Status on Polyunsaturated Fatty Acids and Sleep

To elucidate the relationship between PUFAs and sleep, we conducted a comparative analysis of relevant research data. Animal studies suggest that changes in the composition of long-chain fatty acids within the pineal membrane are associated with diminished melatonin rhythms and an impaired circadian function. Additionally, the intake of long-chain fatty acids significantly influences the regulation of the sleep–wake cycle [9,37]. A diet deficient in n-3 PUFAs has been linked to chronic hyperactivity, disrupted melatonin rhythms, and heightened dopamine activity in hamsters [38]. Additional research reveals a positive correlation between DHA levels in the hippocampus and serotonin content; specifically, a higher DHA intake is associated with increased serotonin concentrations in this brain region [11]. Experiments’ findings demonstrate that sleep deprivation results in reduced levels of various proteins in rats, while fish oil supplementation can restore some of these protein levels [39]. However, it does not fully reverse the social memory deficits caused by sleep deprivation. It is important to note that variations in the species, age, and rearing environments of the animals used across different studies may affect the consistency of the findings.

In human studies, Boone KM et al. examined the effects of DHA and AA on the sleep patterns of preterm infants (*N* = 377, gestational age less than 35 weeks, and aged 10–16 months) [40]. Participants received daily supplementations of 200 mg DHA and 200 mg AA for a duration of 180 days, followed by an average follow-up period of 8 months post-intervention. While no significant overall differences in sleep changes were observed between the supplementation group and the control group, the treatment group demonstrated an increased nighttime sleep duration and reduced sleep issues among male children. Furthermore, significant improvements in sleep quality were noted in children aged 7–12 years following DHA supplementation [41,42]. Conversely, a recent study conducted in Brazil found no significant association between DHA supplementation and sleep quality in extremely premature infants assessed between 12 and 24 months after birth [43]. Additionally, Patan et al. reported that DHA had a beneficial regulatory effect on sleep among healthy young adults compared to the placebo group, leading to improved sleep efficiency, reduced sleep latency, and an increased total sleep time and time spent in bed [16]. In contrast, the effects of EPA were minimal. The variability in these findings may be attributed to differences in study populations, including variations in birth conditions and genetic backgrounds, among preterm infants.

Interestingly, a cross-sectional survey involving 18,310 participants found that the intake of n-6 fatty acids exhibited an inverse U-shaped relationship with the risk of sleep disorders, while the ratio of n-6 to n-3 fatty acids was positively correlated with this risk. Notably, the consumption of both n-3 and n-6 fatty acids was negatively associated with the likelihood of experiencing abnormal sleep duration across various age groups and genders [11]. Research by Judge et al. on maternal DHA intake during pregnancy revealed that infants in the intervention group—who consumed grain bars containing an average of 300 mg of DHA five days a week—experienced significantly fewer awakenings during both quiet and active sleep compared to those in the placebo group [29]. This suggests that DHA supplementation during pregnancy may confer benefits for infant sleep and cognitive development. Additionally, Cheruku et al. found that higher maternal DHA concentrations during pregnancy were associated with more mature sleep patterns in newborns, indicating that prenatal DHA supply could influence brain phospholipids and neural function [29]. Furthermore, ensuring a daily intake of at least 16.1 g of fish or sufficient n-3 PUFAs during pregnancy may lower the risk of infants sleeping less than 11 h by their first birthday. This association likely stems from the positive effects of n-3 PUFAs from fish on infant neurodevelopment [29].

**Figure 2:**
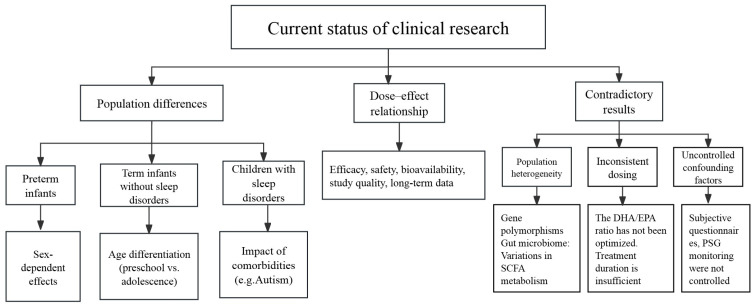
Current research on the relationship between PUFAs and children’s sleep reveals contradictions in the dose–effect relationship, substantial population heterogeneity, and deficiencies in study design. There is an urgent need for high-quality randomized controlled trials to establish the optimal dosage and assess the long-term safety of PUFAs.

In conclusion, the studies reviewed encompass a diverse range of age groups and backgrounds. However, some research lacks rigorous control over dietary factors that may affect sleep, such as daily intake of vitamins and minerals, as well as levels of exercise among participants. This oversight may hinder accurate assessments of the relationship between PUFAs and sleep. Additionally, we have summarized the limitations of PUFAs in population applications, as illustrated in Figure 2.

## 4. Mechanisms by Which Polyunsaturated Fatty Acids Improve Sleep

### 4.1. Neurobiological Mechanisms

Omega-3 fatty acids are essential for the effective functioning of the nervous system and play a significant role in intercellular signaling. Insufficient levels of omega-3 fatty acids can adversely affect the oscillatory activity of cortical neurons during sleep and disrupt the sleep–wake cycle [23,44]. Research indicates that an increased intake of DHA correlates with elevated serotonin levels in the hippocampus, where serotonin is key in preparing, initiating, and maintaining sleep [45,46]. Several studies illustrate that EPA influences sleep by diminishing the production of E2 series prostaglandins and enhancing the release of serotonin from presynaptic neurons. Additionally, DHA enhances the effectiveness of serotonin receptors by improving the fluidity of the postsynaptic neuron cell membrane, thereby optimizing neurotransmission related to sleep regulation [47,48]. In the human gut, microbial colonization leads to the production of various neurotransmitters and cytokines, including short-chain fatty acids, dopamine, gamma-aminobutyric acid (GABA), 5-hydroxytryptophan, and melatonin [49,50]. These metabolites interact with the vagus nerve and influence the central nervous system through enteroendocrine cell modulation. Notably, bacteria such as Lactobacillus and Bifidobacterium can secrete GABA, and GABA deficiencies have been positively associated with sleep disorders. Furthermore, a high intake of DHA can increase its concentration in the pinealocyte cell membrane, fostering melatonin synthesis. Conversely, reduced levels of DHA lead to a decreased melatonin production, which results in poor sleep quality, shorter total sleep time, and extended sleep latency [40,51].

The central oscillator of the circadian rhythm system is found in the suprachiasmatic nucleus of the hypothalamus [52], relying primarily on transcription–translation feedback loops for regulation. These loops are composed of transcription factors derived from clock genes, including Circadian Locomotor Output Cycles Kaput, Brain and Muscle Arnt-like Protein 1, and Period and Cryptochrome [53]. Wefers et al.’s research revealed that disruptions in the circadian rhythm lead to a significant upregulation of long-chain fatty acid expression [54]. DHA and EPA may facilitate a phase advancement of the diet-induced clock molecule Period2 through the GPR120-dependent insulin signaling pathway. Studies employing soybean oil or fish oil enriched with DHA and EPA have shown a marked enhancement in the phase advancement of PER2 expression rhythms [55]. Moreover, DHA can restore oscillations in the circadian rhythm via the insulin-induced gene 2 (INSIG2)-SREBP pathway, which is mediated by clock nuclear receptors retinoic acid receptor-related orphan receptor alpha (RORα) and REV-ERBα, ultimately enhancing the rhythm of lipid metabolism [56].

The biological clock influences not only digestive physiology and intestinal barrier integrity but also the regulation of hormones and peptides that manage food intake, thereby affecting hunger and satiety [57]. Additionally, the microbiota–gut–brain axis impacts circadian rhythms and modulates the brain’s response to melatonin [58]. Diets high in healthy fats—such as monounsaturated fatty acids from olive oil and n-3 PUFAs from fish oil—are beneficial for gut microbiota and can improve sleep quality [59]. Omega-3 PUFAs may affect sleep by altering gut microbiota composition, regulating short-chain fatty acid levels, repairing intestinal and blood–brain barriers, and decreasing pro-inflammatory mediators [60]. Increased fish oil intake has been associated with reduced growth of enterobacteriaceae and higher bifidobacteria abundance, contributing to the gut microbiota balance. Omega-3 fatty acids may also help thicken the intestinal mucosa, enhance gut health, and strengthen mucosal barrier function [61,62]. Furthermore, DHA optimizes the bile acid metabolism by influencing the circadian rhythm of gut microbiota through interactions with INSIG2 and sterol regulatory element-binding protein pathways [63].

### 4.2. Inflammation and Immune Regulation Mechanisms

Poor sleep quality is strongly linked to inflammatory responses [64,65]. Research indicates that insufficient sleep activates inflammatory signaling pathways, such as NF-κB and activator Protein 1, leading to elevated mRNA levels of pro-inflammatory cytokines, including interleukin-6 (IL-6) and tumor necrosis factor-alpha (TNF-α). This increase in cytokine production can further stimulate monocytes to generate more IL-6 and TNF-α in response to TLR4 stimulation [66,67]. Notably, elevated TNF-α levels inhibit the expression of hypothalamic peptides, disrupting sleep homeostasis and resulting in fragmented sleep [68]. In experimental models of OSAHS, there is a significant upregulation of TNF-α expression in both the central nervous system and peripheral tissues. This rise in TNF-α suppresses sympathetic activation in pinealocytes, contributing to prolonged sleep latency and increased nighttime awakenings [69]. Similarly, elevated IL-6 levels can disrupt circadian rhythms and reduce total sleep duration [70]. Patients with OSAHS often have higher serum or plasma levels of IL-6 compared to healthy controls. The upregulation of pro-inflammatory cytokines like IL-6 exacerbates the activation of the NF-κB pathway, worsening sleep conditions. Ultimately, high-quality sleep is associated with lower IL-6 secretion, while increased IL-6 levels may lead to excessive daytime sleepiness [71].

Furthermore, omega-3 fatty acids enhance the transcriptional activity and expression of RORα through Maresin 1, which is produced by 12-lipoxygenase and acts as an endogenous ligand for RORα. This relationship increases macrophage activity and reduces inflammation [72,73]. Additionally, omega-3 fatty acids promote the production of short-chain fatty acids vital for gut health due to their anti-inflammatory properties, potentially improving sleep duration [74].

In contrast, the mechanisms by which dietary omega-6 fatty acids affect sleep disorders are not well understood. It is theorized that omega-6 fatty acids act as precursors to AA, a pro-inflammatory lipid mediator that may elevate the risk of sleep disturbances [27,75,76]. AA is also a precursor for endogenous cannabinoids such as anandamide (AEA) and 2-arachidonoylglycerol. Evidence suggests that AEA may lower airway tension, potentially increasing the risk of respiratory obstruction during sleep [77]. Additionally, elevated plasma concentrations of AEA have been noted in relation to the severity of OSAHS [78]. Research shows that a higher omega-6 to omega-3 fatty acid ratio may lead to decreased omega-3 levels in tissues, including the brain, while raising AA levels derived from omega-6 sources. This imbalance could exacerbate the risk of sleep disorders [23]. Nevertheless, existing studies have not conclusively linked PUFAs intake to sleep duration. One possible reason for this ambiguity is that fatty acids can be metabolized into ketone bodies in the liver, and increased levels of these ketones may stimulate the transcription of the brain-derived neurotrophic factor, influencing sleep duration [79]. Research on animals has demonstrated that LA is less likely to accumulate in the brain tissue of developing rats compared to DHA, even though their rates of brain entry are comparable [80]. LA enhances the activity of AMP-activated protein kinase (AMPK), which is key for activating autophagy and antioxidant systems. A reduction in LA levels may compromise AMPK activity, which could result in fragmented sleep, more frequent awakenings, and shorter sleep duration [81]. Additionally, LA plays a role in preventing oxidative stress in insulin-secreting cells and fibroblasts. If LA levels decrease, increased oxidative stress could cause fluid buildup in the neck and elevate upper airway resistance, potentially triggering or worsening OSAHS [82,83,84].

Recent studies have demonstrated that different types of fatty acids exhibit distinct relationships with lung function [85]. Specifically, α-LA, an upstream fatty acid in the omega-3 metabolic pathway, may have its association with lung function influenced by metabolic efficiency. Conversely, DHA, located at the terminal end of this pathway, consistently correlates with lung function phenotypes. Longitudinal studies reveal that for every 1% increase in DHA, there is a corresponding 1.4 mL reduction in the annual decline of forced expiratory volume in one second (FEV1) and a 2.0 mL reduction in forced vital capacity (FVC) decline, both of which are linked to a decreased incidence of airway obstruction. Moreover, the link between omega-3 fatty acids and lung function is influenced by variables such as gender, ethnicity, and smoking habits.

In summary, the studies mentioned above suggest that PUFAs may affect sleep through mechanisms associated with inflammation and lung function. Omega-3 PUFAs, such as EPA and DHA, are essential in reducing airway and systemic inflammation by lowering levels of TNF-α and IL-6. This reduction not only alleviates upper airway edema but also stabilizes respiratory control centers during sleep. Conversely, elevated concentrations of eicosanoids and endocannabinoids derived from AA, including anandamide, can lead to bronchial hyperreactivity and heighten the risk of upper airway collapse, thereby worsening OSAHS. Consequently, maintaining a balanced PUFA profile that reduces pro-inflammatory mediators while ensuring optimal pulmonary function is anticipated to enhance sleep quality and minimize nocturnal hypoxemia in children.

### 4.3. Endocrine Regulatory Mechanism

The hypothalamic–pituitary–adrenal (HPA) axis serves as a crucial neuroendocrine system in the human body, primarily involved in the regulation of various physiological processes, particularly those related to stress responses. In response to stress, the HPA axis becomes activated, leading the hypothalamus to release the corticotropin-releasing factor (CRF). This triggers the pituitary gland to secrete the adrenocorticotropic hormone (ACTH), which stimulates the adrenal cortex to produce cortisol and other glucocorticoids. These hormones are vital for regulating numerous bodily systems, including metabolism, immune response, and cardiovascular function, thereby supporting the body’s adaptation to stress. Moreover, the HPA axis also plays an integral role in managing circadian rhythms, emotions, cognition, and the sleep–wake cycle, all of which are fundamental for maintaining homeostasis. Alongside the locus coeruleus and the norepinephrine-autonomic nervous system, the HPA axis forms a comprehensive neuroendocrine system that influences alertness and sleep regulation. In this framework, lipocalin-type prostaglandin D2 synthase, predominantly expressed in oligodendrocytes, the arachnoid membrane, and the choroid plexus, is responsible for synthesizing prostaglandin D2. Meanwhile, the DP1 receptor, primarily located in cell membranes adjacent to cerebrospinal fluid and in the key areas governing sleep, is instrumental in this process [86].

A long-term experimental study conducted on rats [87] indicated that n-3 PUFAs supplementation led to substantial reductions in the plasma levels of ACTH and corticosterone, along with a significant decline in CRF expression within the hypothalamus. These findings suggest that n-3 PUFAs effectively mitigate the excessive activation of the HPA axis, aiding in the restoration of its secretion function to normal levels. Moreover, the n-3 PUFA was found to increase glucocorticoid receptor expression in the hippocampus, which is crucial for re-establishing the negative feedback mechanism of the HPA axis and reducing stress-induced dysfunction. Additional results demonstrated lower levels of PGE_2_, TNF-α, and IL-6 in the n-3 PUFA group compared to the control group, providing further evidence that n-3 PUFA regulates the HPA axis by suppressing inflammatory responses, thus showcasing long-term regulatory effects. Notably, both lifelong and post-weaning supplementation with n-3 PUFA significantly elevated DHA levels in the brains of the rats.

In summary, we have prepared Table 2 to elucidate the specific mechanisms by which PUFAs influence children’s sleep. Additionally, we have summarized the molecular characteristics of PUFAs and their roles related to sleep and cognition in Table 3.

## 5. Types of Polyunsaturated Fatty Acid Preparations and Safety in Clinical Studies

Fish oil products on the market are categorized into two main types: preventive and therapeutic formulations. Preventive fish oil supplements generally contain 180 mg of EPA and 120 mg of DHA per 1000 mg, whereas therapeutic formulations consist of fatty acids in the form of ethyl esters, boasting concentrations roughly three times higher than those in preventive supplements. Additionally, therapeutic formulations are also manufactured under rigorous good manufacturing practices, which minimize contamination from impurities, saturated fatty acids, or cholesterol—criteria that are not generally applied to preventive supplements. In terms of bioavailability, the ethyl ester form of fatty acids in therapeutic preparations is absorbed and utilized more effectively by the human body than preventive fish oil supplements [88]. Significant advances have been made in synthesizing medium- and medium-and-long-chain triglycerides enriched with DHA, with Lipozyme RM IM identified as the most efficient lipase. The optimized reaction conditions (6 h reaction time, 8 wt% lipase loading, a 3:1 substrate molar ratio, and 55 °C temperature) revealed that this lipase could be reused up to 17 times. This formulation demonstrates improved digestibility and absorption in infants’ digestive systems, indicating its potential to enhance the bioavailability of DHA in infant formula [89].

Currently, n-3 products are available in three primary forms: ethylated, free, and glycerolized, each possessing unique advantages and disadvantages concerning bioavailability and stability. However, oxidation remains a critical issue for fish oil products [90]. Studies show that sphingoid bases derived from amino compounds can effectively inhibit the formation of volatile compounds during the initial stages of fish oil oxidation when paired with alpha-tocopherol [91]. Recent research has highlighted that microencapsulation is an effective strategy for improving fish oil stability [92]. By transforming fish oil from a liquid to a solid form, microencapsulation establishes a physical barrier that limits exposure to oxygen and moisture, consequently slowing down the oxidation process. Importantly, different drying techniques can significantly affect the oxidative stability of fish oil microcapsules, with spray-freeze-drying proving to be especially effective in preserving their stability.

According to data from the National Health and Nutrition Examination Survey for 2015 to 2016 [93], the average daily intake of n-3 PUFAs among adults is only 100 mg, which includes 70 mg of EPA and DHA. For infants, the generally recommended dosage of DHA ranges from 40 to 120 mg/kg/d [94,95]. It is important to note that while deep-sea fish are rich in DHA, certain species—such as shark, king mackerel, swordfish, and tilefish—may have elevated levels of contaminants like methylmercury and polychlorinated biphenyls. Therefore, it is crucial to consider these risks when seeking to obtain DHA through dietary sources.

Additionally, n-3 fatty acids exhibit potential antithrombotic effects, and alterations in the balance of their metabolites may enhance this action. However, long-term high-dose consumption of n-3 fatty acids, such as DHA, could increase the risk of prolonged bleeding times and may potentially elevate low-density lipoprotein cholesterol levels. In individuals with non-insulin-dependent diabetes, the intake of n-3 fatty acids may negatively impact blood glucose control [96]. Thus, the appropriate timing and dosage of n-3 fatty acid supplementation require further validation through comprehensive clinical and animal studies.

A recent systematic review and meta-analysis explored the safety and tolerability of n-3 PUFAs supplements [97]. The findings indicated that no serious adverse events related to n-3 PUFAs were reported, However, users may experience some potential mild adverse effects. Specifically, non-serious adverse events associated with n-3 PUFAs included an increased incidence of diarrhea and taste disturbances, while a bleeding tendency was linked to prescription omega-3 PUFAs (RxOME3FAs). In terms of lipid profiles, n-3 PUFAs demonstrated beneficial effects on non-high-density lipoprotein, total cholesterol, triglycerides, and very low-density lipoprotein, with RxOME3FAs exhibiting an even greater efficacy. Regarding non-lipid laboratory indicators, n-3 PUFAs were found to lower levels of alkaline phosphatase and C-reactive protein, while they increased levels of alanine aminotransferase and blood urea nitrogen. Additionally, studies evaluating routine adverse events associated with RxOME3FAs reported elevated fasting blood glucose and glycated hemoglobin levels. However, the generalizability of these results is constrained, as the study population primarily included middle-aged individuals with dyslipidemia, cardiovascular diseases, and type 2 diabetes. Furthermore, there is insufficient data regarding bleeding tendencies, and the randomized controlled trial samples were highly selective, which may have led to the omission of rare or long-term adverse events. Currently, no specific systematic review exists on the adverse reactions associated with PUFAs supplementation in children.

**Figure 3:**
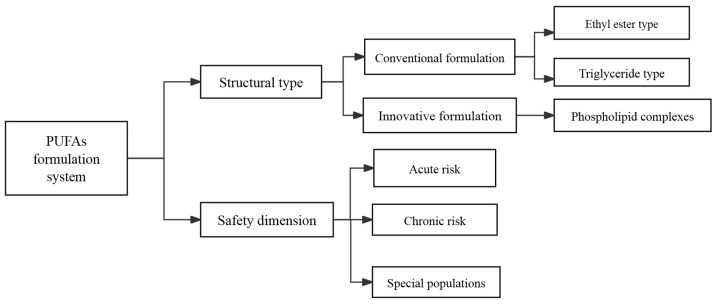
At present, there are three available formulations of PUFAs, ethyl esters, triglycerides, and phospholipid complexes. Nonetheless, safety concerns must be carefully considered when utilizing these formulations to enhance sleep. Important factors include the possibility of acute tolerance reactions, including gastrointestinal disturbances, as well as chronic risks such as bleeding and effects on blood glucose and lipid profiles. This is particularly significant for at-risk groups, such as premature infants and children with diabetes or obesity, who may experience a greater likelihood of adverse effects.

Accordingly, we have included a figure that delineates the principal formulations of PUFAs and their respective safety profiles, as shown in Figure 3.

## 6. Conclusions and Perspectives

Sleep is a complex, multifactorial phenomenon, and recent studies suggest that PUFAs can influence its duration and quality. Both DHA and EPA seem to enhance pediatric sleep by regulating neurotransmitters, activating anti-inflammatory pathways, and stabilizing circadian rhythms. In contrast, overconsumption of C20:4 or LA may worsen inflammation and disrupt sleep architecture. However, there remain considerable gaps in our knowledge regarding these relationships. Although the findings are promising, several obstacles must be overcome before PUFAs can be consistently recommended for enhancing children’s sleep.

The optimal daily dosage and the appropriate ratio of EPA to DHA are still uncertain. Pediatric trials have reported administering DHA in doses between 40 and 120 mg/kg/day, but these figures primarily stem from studies on infant neurodevelopment [94,95]. In addition, there is a scarcity of data regarding the effects of supplementation beyond 6 to 12 months, leaving potential impacts on bleeding time and glycemic control in children with comorbid metabolic disorders inadequately investigated [96,97]. Furthermore, many studies suffer from insufficient power and fail to stratify participants by genetic polymorphisms related to fatty-acid-metabolizing enzymes (e.g., FADS1/2) or variations in the gut microbiome composition, factors known to affect PUFA conversion and clinical effectiveness [23].

Lastly, the predominant use of subjective sleep questionnaires presents a significant limitation in the literature, with objective assessments like polysomnography (PSG) and actigraphy being infrequently applied. Additionally, dietary confounders such as micronutrients, physical activity, and screen time are often not adequately controlled in these investigations.

To advance the field, future studies should embrace precision nutrition methodologies that integrate plasma PUFA metabolomics and gut microbiome profiling to pinpoint genuine responders. In addition, it is essential to conduct multi-center, double-blind, dose-escalation randomized controlled trials utilizing objective measures of sleep, such as actigraphy and polysomnography. Follow-up periods of no less than 12 months are necessary to thoroughly assess safety and efficacy in diverse pediatric cohorts.

**Figure 4:**
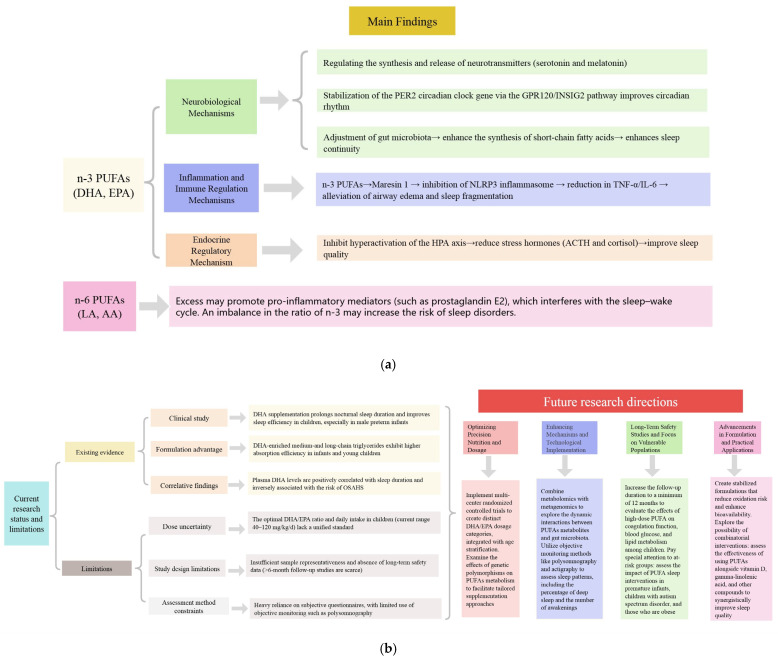
(**a**) Key findings in the research. (**b**) Current limitations in the research and directions for future research.

Until high-quality evidence becomes available, clinicians may cautiously consider recommending omega-3-rich fish or microencapsulated DHA supplements for children experiencing persistent sleep disturbances and documented low omega-3 status, while closely monitoring for tolerability issues such as bleeding and gastrointestinal effects [89]. Innovative formulations of medium- and long-chain triglycerides enriched with DHA enhance palatability and gastrointestinal absorption in toddlers [89] and could serve as a viable first-line option in pediatric sleep clinics. Ultimately, PUFA interventions should be incorporated into multidisciplinary sleep health programs that also target lifestyle factors, such as light exposure and physical activity, requiring collaboration among nutritionists, sleep physicians, psychologists, and immunologists. To this end, we summarize the manuscript’s main findings and outline future research endeavors based on these findings (Figure 4a,b).

## Figures and Tables

**Table 1:** Types, sources, and functions of PUFAs.

Type	Main Components	Sources	Functions	Relationship with Health
n-3	DHA, EPA	Marine fish, such as mackerel, salmon, herring, and sardines	Involved in processes such as synaptogenesis, gene expression, neuroplasticity, cell membrane fluidity, neurotransmitter transmission, and myelination, which are crucial for the functioning of the nervous system	Helps to reduce the risk of chronic inflammation-related diseases in the brain and enhances cognitive function
n-6	LA AA	Soybean oil, corn oil, sunflower oil, peanut oil, meat, eggs, and dairy	Plays an important role in regulating inflammatory responses, with its metabolites involved in various physiological processes	Under pathological conditions such as oxidative stress, an excess of AA is linked to the development of cardiovascular and metabolic diseases

**Table 2:** Mechanisms by which PUFAs regulate sleep.

Type	Mechanism Classification	Specific Pathways	Effects on Children’s Sleep
Omega-3 series	Neurobiological Mechanisms—Regulation of Neurotransmitters	DHA increases the concentration of serotonin in the hippocampus EPA reduces E2 series prostaglandins and promotes serotonin release DHA optimizes serotonin receptor function	Contributes to the preparation, initiation, and maintenance of sleep; a deficiency may negatively impact the oscillatory activity of cortical neurons during sleep and the sleep–wake cycle
	Neurobiological Mechanisms—Effects on Circadian Rhythm	DHA and EPA advance the molecular phase of PER2 through the GPR120-dependent insulin signaling pathway DHA restores circadian rhythm via the INSIG2-SREBP pathway mediated by RORα and REV-ERBα	Reduced levels of DHA can lead to poor sleep quality and a shorter total sleep duration
	Neurobiological Mechanisms—Gut–Brain Axis	High DHA levels increase the content of DHA in the pineal cell membranes, promoting melatonin synthesis A healthy fat diet influences the gut microbiome to enhance sleep Omega-3 polyunsaturated fatty acids alter the composition of the gut microbiome and the concentration of short-chain fatty acids	Regulating circadian rhythms, improving lipid metabolism, and supporting gut health, thereby promoting sleep
	Inflammation and Immune Regulatory Mechanisms—Anti-Inflammatory Effects	Omega-3 fatty acids generate Maresin1, which enhances RORα activity and reduces inflammation Omega-3 fatty acids promote the production of short-chain fatty acids, supporting gut health	Reducing inflammation’s interference with sleep may positively impact sleep duration
	Inflammation and Immune Regulatory Mechanisms—Interaction Between Sleep and Inflammation	Insufficient sleep activates inflammatory signaling pathways, increasing IL-6 and TNF-α levels; TNF-α suppresses the expression of hypothalamic peptide hormones, impacting sleep homeostasis IL-6 leads to disruptions in the circadian rhythm, resulting in a shorter total sleep duration	Affecting circadian rhythms and reducing total sleep duration
	Endocrine Regulatory Mechanisms	Supplementation with n-3-PUFA lowers plasma levels of ACTH and corticosterone n-3-PUFA increases the expression of glucocorticoid receptors in the hippocampus, restoring HPA axis negative feedback	Inhibiting the excessive activation of the HPA axis alleviates stress-induced dysregulation of HPA axis function, thereby regulating sleep
Omega-6 series	Inflammation and Immune Regulatory Mechanisms	It is a precursor to prostaglandin-like substances, and arachidonic acid acts as a precursor for endogenous cannabinoids; related metabolites may increase the risk of respiratory obstruction during sleep An elevated n-6:n-3 ratio increases the risk of sleep disorders	Lead to sleep disturbances, the intrinsic mechanisms by which dietary n-6 fatty acids contribute to sleep disorders are still unclear
		LA can enhance AMP kinase activity, activating autophagy and the antioxidant system A decrease in LA levels may lead to sleep fragmentation, increased awakenings, and shorter sleep duration A decline in LA levels may trigger fluid accumulation in the neck and increased upper airway resistance, leading to OSAHS and worsening the condition	Affect the continuity and quality of sleep

**Table 3:** Molecular characteristics and sleep–cognition-related effects of key PUFAs.

PUFAs	Molecular Formula	Simplified Structure	Key Sleep–Cognition Benefits	References
DHA	C_22_H_32_O_2_	CH_3_-CH_2_-CH=CH-CH_2_-CH=CH-CH_2_-CH=CH-CH_2_-CH=CH-CH_2_-CH=CH-CH_2_-CH=CH-CH_2_-CH_2_-COOH	DHA is associated with increased levels of serotonin and melatonin, reduced risk of OSAHS, and improved sleep efficiency.	[9,10,11,41,42]
EPA	C_20_H_30_O_2_	CH_3_-CH_2_-CH=CH-CH_2_--CH=CH-CH_2_-CH=CH-CH_2_-CH=CH-CH_2_-CH=CH-CH_2_-CH_2_-COOH	EPA is associated with decreased levels of TNF-α and IL-6, stabilization of the circadian rhythm, and improved sleep duration.	[7,10,11]
α-LA	C_18_H_30_O_2_	CH_3_-(CH_2_-CH=CH)_3_--(CH_2_),-COOH	Precursor to EPA and DHA, limited direct sleep evidence.	[19,21]
LA	C_18_H_32_O_2_	CH_3_-(CH_2_)_4_-CH=CH-CH_2_-CH=CH-(CH_2_),-COOH	A balanced intake of LA is necessary, as excessive consumption may promote inflammation and disrupt sleep.	[22,26]
AA	C_20_H_32_O_2_	CH_3_-(CH_2_)_4_-CH=CH-CH_2_-CH=CH-CH_2_-CH=CH-CH_2_-CH=CH-(CH_2_)_3_-COOH	AA serves as a precursor to pro-inflammatory eicosanoids, and elevated levels of AA are associated with poor sleep.	[22,77,78]

## Abbreviations

Abbreviations’	Full Terms
PUFAs	Polyunsaturated fatty acids
DHA	Docosahexaenoic acid
OSAHS	Obstructive sleep apnea hypopnea syndrome
EPA	Eicosapentaenoic acid
α-LA	Alpha-linolenic acid
LA	Linoleic acid
AA	Arachidonic acid
GABA	Gamma-aminobutyric acid
INSIG2	Insulin-induced gene 2
RORα	Retinoic acid-related orphan receptor alpha
IL-6	Interleukin-6
TNF-α	Tumor necrosis factor-alpha
AEA	Anandamide
AMPK	AMP-activated protein kinase
HPA	Hypothalamic-pituitary-adrenal
CRF	Corticotropin-releasing factor
ACTH	Adrenocorticotropic hormone
RxOME3FAs	Prescription omega-3 polyunsaturated fatty acids
PSG	Polysomnography
